# The impact of orthodontic-surgical treatment on facial expressions—a four-dimensional clinical trial

**DOI:** 10.1007/s00784-023-05195-9

**Published:** 2023-08-10

**Authors:** Anja Quast, Monika Sadlonova, Thomas Asendorf, Carlotta Derad, Jérémy Mouchoux, Julia Horn, Henning Schliephake, Philipp Kauffmann, Philipp Meyer-Marcotty

**Affiliations:** 1 Department of Orthodontics, University Medical Center Goettingen, Robert-Koch-Str. 40, 37075 Goettingen, Germany; 2 Department of Psychiatry, Massachusetts General Hospital, Boston, MA USA; 3 Department of Psychiatry, Harvard Medical School, Boston, MA USA; 4 Department of Psychosomatic Medicine and Psychotherapy, University of Goettingen Medical Center, Goettingen, Germany; 5 Department of Cardiovascular and Thoracic Surgery, University of Goettingen Medical Center, Goettingen, Germany; 6 Department of Medical Statistics, University Medical Center Goettingen, Goettingen, Germany; 7 Department of Oral and Maxillofacial Surgery, University Medical Center Goettingen, Goettingen, Germany

**Keywords:** Facial motion, Stereophotogrammetry, Orthognathic surgery, Quality of life, Self-efficacy, Dentofacial deformity

## Abstract

**Objective:**

The objective of this clinical trial was to compare facial expressions (magnitude, shape change, time, and symmetry) before (T0) and after (T1) orthognathic surgery by implementing a novel method of four-dimensional (4D) motion capture analysis, known as videostereophotogrammetry, in orthodontics.

**Methods:**

This prospective, single-centre, single-arm trial included a total of 26 adult patients (mean age 28.4 years; skeletal class II: *n* = 13, skeletal class III: *n* = 13) with indication for orthodontic-surgical treatment. Two reproducible facial expressions (maximum smile, lip purse) were captured at T0 and T1 by videostereophotogrammetry as 4D face scan. The magnitude, shape change, symmetry, and time of the facial movements were analysed. The motion changes were analysed in dependence of skeletal class and surgical movements.

**Results:**

4D motion capture analysis was feasible in all cases. The magnitude of the expression maximum smile increased from 15.24 to 17.27 mm (*p* = 0.002), while that of the expression lip purse decreased from 9.34 to 8.31 mm (*p* = 0.01). Shape change, symmetry, and time of the facial movements did not differ significantly pre- and postsurgical. The changes in facial movements following orthodontic-surgical treatment were observed independently of skeletal class and surgical movements.

**Conclusions:**

Orthodontic-surgical treatment not only affects static soft tissue but also soft tissue dynamics while smiling or lip pursing.

**Clinical relevance:**

To achieve comprehensive orthodontic treatment plans, the integration of facial dynamics via videostereophotogrammetry provides a promising approach in diagnostics.

**Trial registration number:**

DRKS00017206.

**Supplementary Information:**

The online version contains supplementary material available at 10.1007/s00784-023-05195-9.

## Introduction

Facial expressions as a type of nonverbal communication play an essential role in daily conversations. When assessing emotions, facial expressions carry more weight than the spoken message [1, 2]. As early as 1872, Charles Darwin postulated the universality of emotional facial expressions across cultural boundaries in his work “The expression of the emotions in man and animals” [3]. However, the extent of the facial movements can vary significantly between individuals depending on the facial morphology [4–6]. This is also evident in patients with dentofacial deformities, whose movement patterns with certain facial expressions deviate from those of individuals with neutral jaw relation [7]. Orthodontic-surgical treatment seems to standardise facial expressions [7, 8]. Despite these findings, the evaluation of facial movements is not yet a routinely used parameter in orthodontics. Therefore, asymmetries and abnormalities that manifest during facial expressions may remain undetected [9, 10].

Since orthodontic treatment, and in particular orthodontic-surgical treatment, always affects the appearance of the facial soft tissue, three-dimensional (3D) imaging using stereophotogrammetry has become increasingly important in recent years. In orthodontic-surgical treatment, this helps to predict postsurgical soft tissue changes and to improve the surgical plan [11]. However, the surgical effects in the areas of the lower face and the lips, which are very dynamic structures in daily communication, can only be predicted to a limited extent [12–14].

Non-invasive, four-dimensional (4D) video stereophotogrammetry offers a modern and promising approach for the objective recording and evaluation of facial movements [15–17], which has hardly been used in orthodontics to date. This technology allows to record a sequence of 60 3D images with stepwise changes in facial expressions and a radius of 180 to 360° depending on the number and orientation of the cameras [18]. Therefore, after implementing 3D imaging into orthodontic-surgical treatment, the next step is the integration of 4D motion capture.

The aim of the present study was to compare facial expressions before (T0) and after (T1) orthognathic surgery by implementing the novel method of 4D videostereophotogrammetry in orthodontics. We hypothesised that the magnitude, shape change, symmetry, and time of facial expressions change from T0 until T1.

## Subjects and methods

### Trial design

This study was a prospective, single-centre, single-arm trial and investigated facial expressions before (T0) and 4 months after orthognathic surgery (T1). It was approved by the institutional ethics committee (application number 13/2/19) in accordance with the Declaration of Helsinki. All participants gave written informed consent to take part in the study. The trial was registered before recruitment started (DRKS00017206). No changes occurred after trial commencement.

### Participants, eligibility criteria, and settings

Patients seeking orthodontic-surgical treatment with an orthodontics first approach at the Department of Orthodontics, University Medical Center Goettingen, Germany, were enrolled consecutively. The eligibility criteria for participants were adults over 18 years old, orthodontic therapy with fixed appliance, pronounced malocclusion (skeletal class II: Wits appraisal > 2 mm; skeletal class III: Wits-appraisal <  − 2 mm), and indication for combined orthodontic-surgical treatment (classified as grade 4 or higher using the index of orthognathic functional treatment need [19]). Patients with cleft lip and/or palate, craniofacial syndromes, impaired facial motion, previous orthognathic surgery, Menton deviation > 4 mm, or beard were excluded.

### Sample size

As this trial is an early clinical trial with the aim to implement the novel method of 4D videostereophotogrammetry, sample size calculation was not based on a priori-hypothesis testing. Based on previous treatment numbers, in total 45 patients (1 skeletal class I, 21 skeletal class II, 23 skeletal class III) were operated in an interval similar to the scheduled recruitment phase of this study; a feasible sample size of 20 patients was planned for recruitment. The final sample size was 26 patients (13 skeletal class II, 13 skeletal class III) and a complete case analysis was performed.

### Intervention

All patients underwent virtually planned, splint-based orthognathic surgery performed by an interdisciplinary team of orthodontists and maxillofacial surgeons as described previously [20]. The surgical procedure included the preservation of a pre-surgical defined condylar position using a centric splint [21–23]. In bimaxillary surgeries, Le Fort I osteotomy was performed first, followed by bilateral sagittal split osteotomy according to well-recognised protocols [24–27]. All patients were invited to record their facial expressions during smiling and lip purse at baseline (T0, after orthodontic decompensation, 1 to 6 weeks before surgery) and 4 months post-surgical (T1).

### Blinding

Blinding was not applicable.

### Outcome—facial expressions

**Fig. 1:**
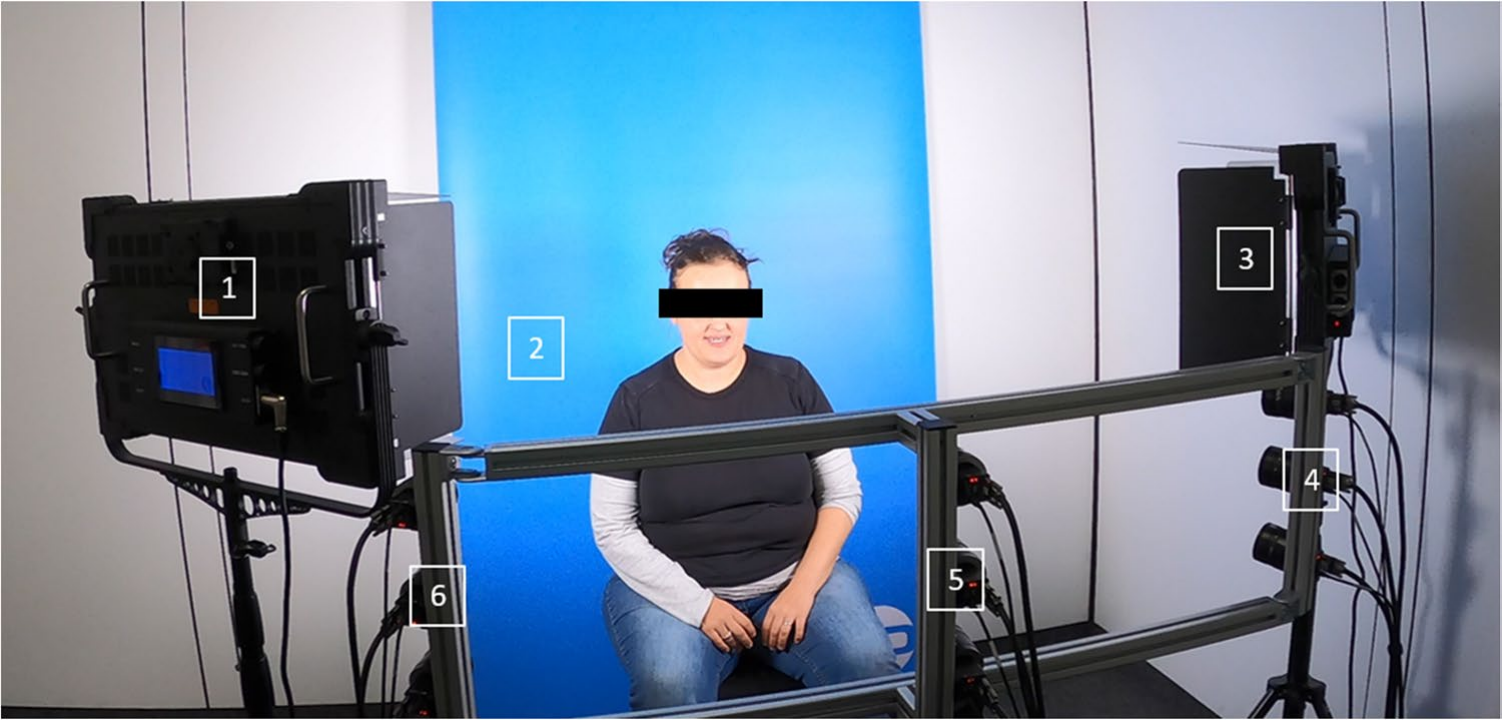
Motion capture system recording facial expressions as 3D videos using non-invasive 4D stereophotogrammetry with nine cameras (6 greyscaled and 3 coloured) from three directions (DI4D PRO System, Dimensional Imaging Ltd., Glasgow, UK). [1] and [3], video lighting system; [2] blue screen; [4, 5], and [6] camera pods

Facial expressions were recorded as 3D videos using non-invasive 4D stereophotogrammetry with nine cameras (6 greyscaled and 3 coloured) and 60 frames per seconds (see Fig. 1; DI4D PRO System, Dimensional Imaging Ltd., Glasgow, UK). Before each capture, the system was calibrated according to a standardised protocol. Each patient was asked to sit upright approximately 95 cm in front of the cameras, to keep the eyes open, to have the head in natural head position and to move the head as little as possible. Then, the patients performed two reproducible facial expressions: (1) maximum smile and (2) lip purse. Both were demonstrated to the patients and practiced several times prior video acquisition.

**Fig. 2:**
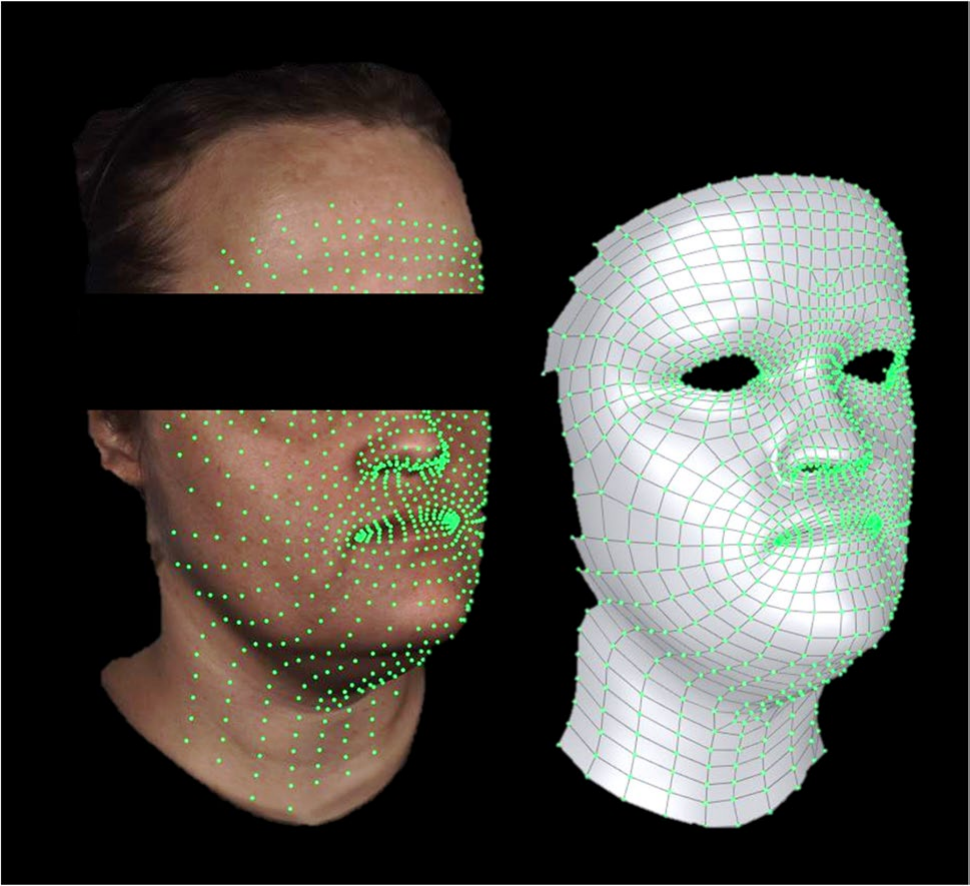
Information captured by videostereophotogrammetry in the first frame. Left side: video data containing the colour and texture of the subject. Right side: dummy mesh applied to the individual patient morphology to track the facial movement

**Fig. 3:**
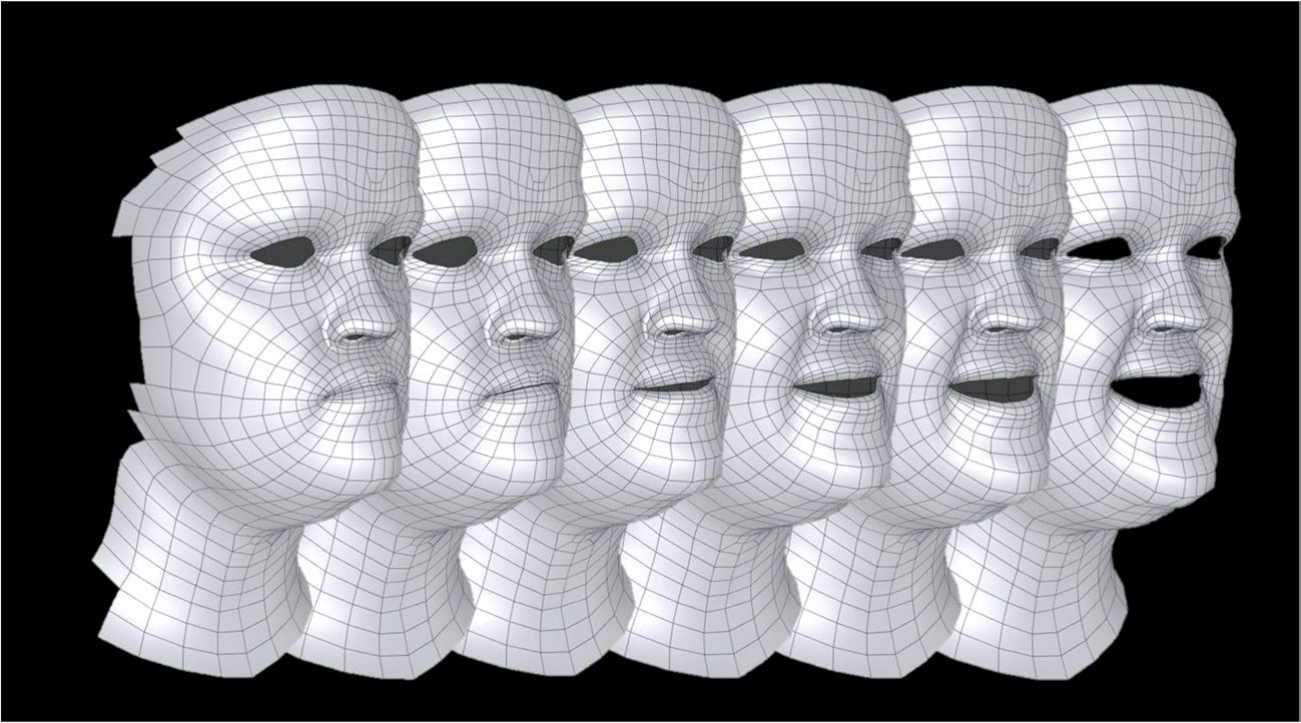
Sequence of exemplary 3D images recorded by the 4D system. The patient-specific mesh applied in the first frame was automatically tracked throughout the sequence of 3D images and manually corrected if necessary

All video captures and post-processing were performed by the same trained investigator (J.H.). The 3D videos were cut to analyse the movements from the last frame in rest position (= start frame) to the first frame with the facial expression at its maximum extent (= end frame) (DI4D Setup, Dimensional Imaging Ltd., Glasgow, UK). To track the facial movement, a dummy mesh was applied to the individual patient morphology in the first frame of each video (see Fig. 2). This patient characteristic mesh was automatically tracked throughout the sequence of 3D images and manually corrected if necessary (see Fig. 3; DI4D track, Dimensional Imaging Ltd., Glasgow, UK). The Euclidean distance *d* between the points of the start frame *s* and the end frame *e* in the 3D space were calculated using Python and a self-edited SciPy based code [28].

**Fig. 4:**
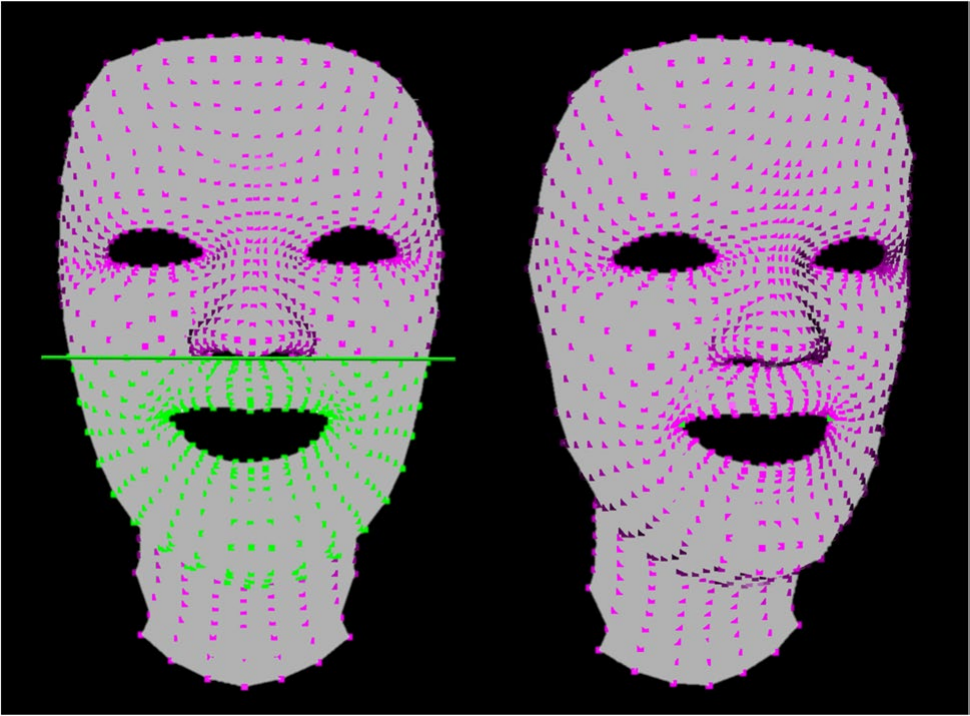
Data analysis in Python. Left side: selection of the region of interest “lower face” limited by a plane through subnasale (green points). The region of interest was determined in the first patient analysed and automatically transferred to each subsequent patient. Right side: the landmarks undergoing the biggest change during facial expression (green points) were determined individually for each capture

Two regions of interest were determined (see Fig. 4):

- the lower face: the area of the lower face limited by a plane through the landmark subnasale parallel to Frankfort Horizontal containing the same automatically selected 559 points for each participant, and
- the landmarks undergoing the biggest change during facial expression: cheilion left and right for maximum smile, and labrale superius and inferius for lip purse.

**Fig. 5:**
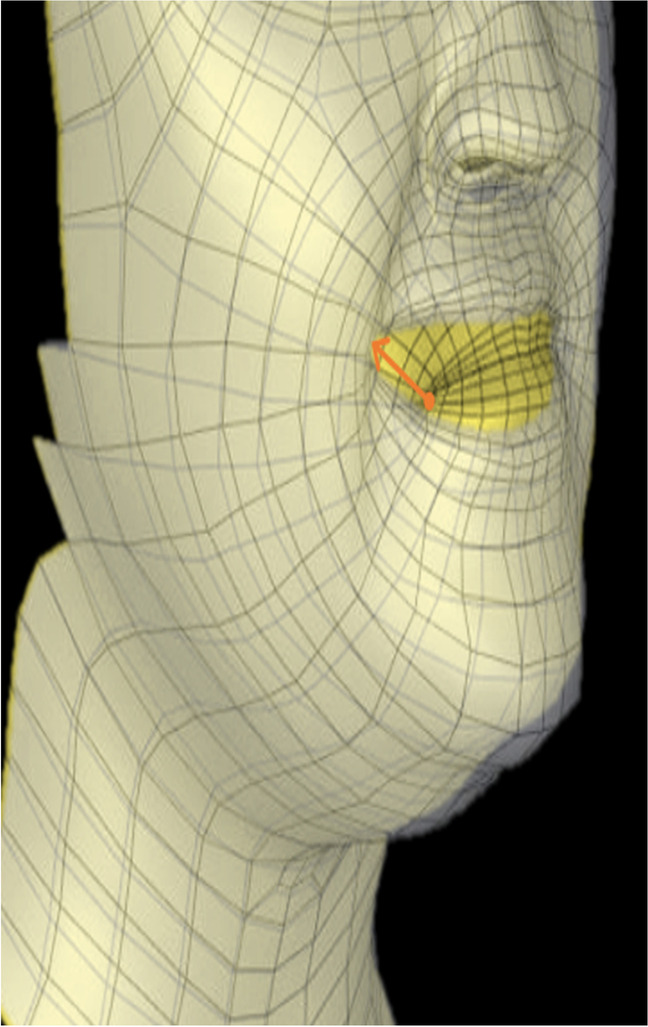
Exemplary illustration of the measurement “magnitude” for the facial expression maximum smile at the landmark cheilion right. The image at rest position (yellow) and the image at the maximum extent of the movement (transparent) are superimposed and the Euclidian distance between the landmark cheilion right at rest position and at maximum smile is measured (orange arrow)

For each facial expression, four parameters were calculated to objectively evaluate the movement (see Table 1 and Fig. 5).

**Table 1:** Parameters calculated to objectively evaluate the facial expressions

Outcome	Definition
Magnitude, mm	Magnitude of facial expression in the most moving area of the lips For maximum smile: $magnitude= \frac{{ d}_{Cheilion right}+{d}_{Cheilion left}}{2}$ For lip purse: $magnitude= \frac{{ d}_{Labrale superius}+{d}_{Labrale inferius}}{2}$
Shape change, mm	Shape change in the lower face during the facial expression calculated as Procrustes distance after superimposing the 3D image of the start and end frame by translating, scaling and rotating as the square root of the sum of squared differences in the positions of all landmarks of the lower face
Symmetry, mm	Differences in facial expression between the two most moving areas of the lips For maximum smile: $symmetry= \left|{d}_{Cheilion right}-{d}_{Cheilion left}\right|$ For lip purse: $symmetry= \left|{d}_{Labrale superius}-{d}_{Labrale inferius}\right|$
Time, s	Time needed to reach the maximum facial expression

d, Euclidian distance

### Method error analysis

To assess the reliability of the 4D tracking of facial expressions, the same investigator repeated the application of the dummy mesh to the individual patients’ morphology in ten cases. Additionally, a second examiner performed the same task one time. Agreement was excellent for the magnitude of facial expression (ICC = 0.982; 95% CI [0.933, 0.995]) and good to excellent for the shape change in the region of interest lower face (ICC = 0.947; 95% CI [0.814, 0.986]).

### Statistical analysis

The analysis was performed using linear mixed-effects models with factors time, skeletal class, and their interaction. A sensitivity analysis using a paired Wilcoxon-signed rank test was performed. Estimated marginal means are reported with 95% confidence interval and compared between time and skeletal class [29]. Pre- and postoperative differences of 4D tracking variables were correlated within a multivariate linear model. Intra-class-correlation (ICC) was calculated to assess agreement between raters and consistency within raters. Baseline data are described using means and standard deviation. Statistical analysis was done using the statistical programming language R 4.1.3 [30].

## Results

### Participant flow and recruitment

**Fig. 6:**
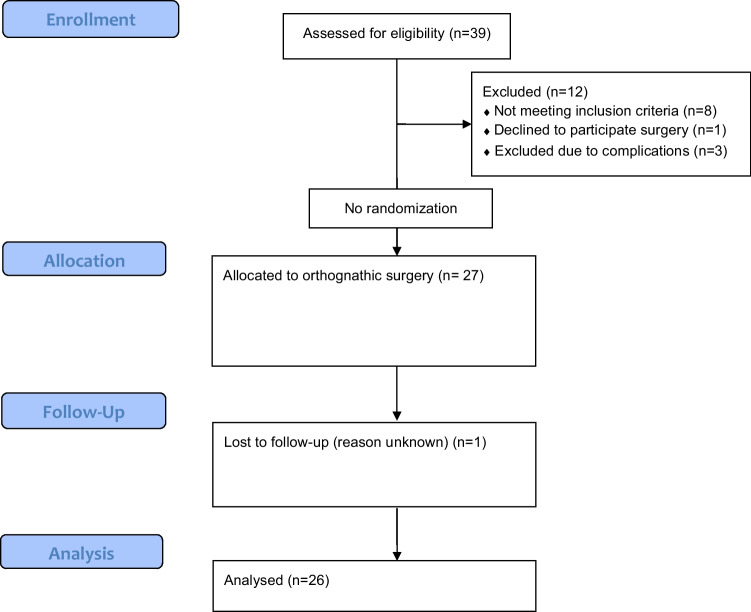
Consort flow chart

Recruitment lasted from September 2020 until August 2021. The last follow-up was completed in December 2021. Of a total of 39 consecutive patients assessed for eligibility, 31 fulfilled the inclusion criteria (see CONSORT flow diagram in Fig. 6). During the observation, 1 patient declined surgery, 1 patient missed his follow-up appointment, 2 patients had surgical complications, and 1 patient was still exceptionally swollen at T1. The final sample consisted of 26 participants. Table 2 shows their baseline demographic data and clinical characteristics.

**Table 2:** Baseline demographic data and clinical characteristics of the final sample (*n* = 26)

Gender	
Female	***n*** = 15
Male	***n*** = 11
Age, years	*M* = 28.4 SD = 8
Skeletal class II Wits appraisal, mm	*n* = 13 *M* = 5.2 SD = 1.6
Skeletal class III Wits appraisal, mm	*n* = 13 *M* = -9.2 SD = 4.4
Surgical intervention Le Fort I + BSSO BSSO	*n* = 21 *n* = 5
Surgical movements Sagittal translation maxilla, mm	*M* = 2.82 SD = 2.2
Vertical translation maxilla, mm	*M* = 1.76 SD = 1.3
Transversal translation maxilla, mm	*M* = 0.95 SD = 0.9
Sagittal translation mandibula, mm	*M* = 4.13 SD = 2.83
Vertical translation mandibula, mm	*M* = 2.42 SD = 1.58
Transversal translation mandibula, mm	*M* = 1.48 SD = 1.27
Roll maxillomandibular complex, °	*M* = 0.62 SD = 0.85
Pitch maxillomandibular complex, °	*M* = 1.58 SD = 2.48
Yaw maxillomandibular complex, °	*M* = 0.73 SD = 1.04

*M*, mean; *SD*, standard deviation

### 4D motion capture

4D motion capture was feasible using stereophotogrammetry and resulted in 3D videos with a rate of 60 frames per second. Colour and texture were recorded as well as surface geometry (see Supplementary Information 1 for a video example). All movements could be tracked from the start frame (rest position) to the end frame (maximum movement) semi-automatically.

### Facial expressions

For maximum smile the mean magnitude of movement increased significantly from T0 until T1 by 2 mm. At T1, smiling became more asymmetric. No differences in shape change or time was observed. The mean magnitude of lip purse decreased from T0 until T1 by 1 mm. Shape change, symmetry, and time for the facial expression did not differ significantly between T0 and T1 (Table 3).

**Table 3:** Means and 95% CI of the parameters analysing facial expressions pre- (T0) and post-surgical (T1) in all patients (*n* = 26)

	Pre-surgical T0			Post-surgical T1			
		95% CI			95% CI		
	Mean	Lower	Upper	Mean	Lower	Upper	*p*
Maximum smile							
Magnitude, mm	15.24	13.07	17.4	17.27	15.1	19.44	0.002
Shape change, mm	0.03	0.01	0.05	0.05	0.03	0.07	0.122
Symmetry, mm	0.99	0.52	1.46	1.78	1.31	2.25	0.024
Time, s	0.41	0.36	0.45	0.43	0.39	0.47	0.41
Lip purse							
Magnitude, mm	9.34	8.43	10.25	8.31	7.4	9.22	0.01
Shape change, mm	0.022	0.019	0.026	0.02	0.017	0.024	0.19
Symmetry, mm	1.27	0.88	1.66	1.06	0.67	1.45	0.38
Time, s	0.36	0.33	0.39	0.32	0.29	0.35	0.052

The post-surgical increase in the magnitude of maximum smile and the decrease in the magnitude of lip purse were observed in all but 5 and 6 patients, respectively.

Subgroup analysis showed that these results apply to patients with skeletal classes II and III (Table 4).

**Table 4:** Means and 95% CI in the changes of the facial expressions (T1–T0) compared between patients with skeletal class II (*n* = 13) and skeletal class III (*n* = 13)

	Pre-surgical T0			Post-surgical T1			
		95% CI			95% CI		
	Mean	Lower	Upper	Mean	Lower	Upper	*p*
Skeletal class II Maximum smile							
Magnitude, mm	15.67	12.61	18.74	17.67	14.61	20.73	0.1
Shape change, mm	0.031	0.002	0.06	0.067	0.04	0.097	0.23
Symmetry, mm	0.89	0.23	1.55	1.78	1.12	2.44	0.25
Time, s	0.42	0.36	0.48	0.42	0.36	0.48	1
Skeletal class III Maximum smile							
Magnitude, mm	14.8	11.74	17.87	16.87	13.8	19.93	0.09
Shape change, mm	0.032	0.003	0.062	0.308	0.009	0.067	0.99
Symmetry, mm	1.09	0.42	1.75	1.78	1.12	2.45	0.46
Time, s	0.39	0.33	0.45	0.44	0.38	0.49	0.58
Skeletal class II Lip purse							
Magnitude, mm	8.76	7.47	10.04	7.67	6.38	8.95	0.18
Shape change, mm	0.02	0.01	0.025	0.02	0.012	0.023	0.77
Symmetry, mm	1.22	0.67	1.77	1.32	0.77	1.87	0.99
Time, s	0.38	0.34	0.42	0.31	0.27	0.36	0.12
Skeletal class III Lip purse							
Magnitude, mm	9.92	8.63	11.2	8.96	7.67	10.24	0.27
Shape change, mm	0.025	0.02	0.03	0.024	0.018	0.029	0.79
Symmetry, mm	1.31	0.76	1.87	0.8	0.25	1.35	0.42
Time, s	0.34	0.3	0.39	0.32	0.28	0.37	0.94

The results of the multivariate linear model with the difference in the magnitude of maximum smile as dependent variable showed that the pre- and postsurgical differences in the magnitude of smile cannot be explained by the surgical movements, the type of surgical intervention, or the skeletal class (Table 5). The multivariate linear model for the magnitude of lip purse indicated the same—the postsurgical change in the movement of the lips showed no correlation with the surgical intervention or the skeletal class (Table 6).

**Table 5:** Results of the multivariate linear model with the difference in the magnitude of maximum smile as dependent variable and the surgical movements, the type of surgical intervention, and the skeletal class as explaining variables. The difference in the magnitude of maximum smile cannot be explained by the explaining variables

	Estimate	Std. error	*t* value	*p*
(Intercept)	4.53	3.15	1.44	0.173
Sagittal translation maxilla	0.23	0.48	0.48	0.637
Vertical translation maxilla	− 0.29	0.79	− 0.37	0.719
Transversal translation maxilla	− 0.54	1.14	− 0.47	0.645
Sagittal translation mandibula	− 0.09	0.34	− 0.27	0.793
Vertical translation mandibula	− 0.27	0.53	− 0.51	0.619
Transversal translation mandibula	− 0.21	0.77	− 0.27	0.792
Roll maxillomandibular complex	0.54	0.97	0.55	0.588
Pitch maxillomandibular complex	0.61	0.5	1.22	0.242
Yaw maxillomandibular complex	− 1.36	0.99	− 1.37	0.191
Skeletal class	− 0.47	1.54	− 0.30	0.766
Type of surgical intervention	− 4.43	2.16	− 2.04	0.060

**Table 6:** Results of the multivariate linear model with the difference in the magnitude of lip purse as dependent variable and the surgical movements, the type of surgical intervention, and the skeletal class as explaining variables. The difference in the magnitude of lip purse cannot be explained by the explaining variables

	Estimate	Std. error	*t* value	*p*
(Intercept)	− 1.92	2.41	− 0.8	0.438
Sagittal translation maxilla	0.22	0.37	0.61	0.553
Vertical translation maxilla	− 0.39	0.61	− 0.64	0.534
Transversal translation maxilla	− 0.73	0.87	− 0.84	0.416
Sagittal translation mandibula	− 0.08	0.27	− 0.29	0.775
Vertical translation mandibula	0.37	0.41	0.91	0.383
Transversal translation mandibula	0.19	0.59	0.33	0.746
Roll maxillomandibular complex	0.91	0.74	1.23	0.239
Pitch maxillomandibular complex	0.18	0.38	0.48	0.639
Yaw maxillomandibular complex	− 0.22	0.76	− 0.29	0.776
Skeletal class	− 0.04	1.17	− 0.03	0.977
Type of surgical intervention	0.63	1.65	0.38	0.707

### Harms

No harms were observed.

## Discussion

### Main findings in context of the existing evidence—method

The main strength of this study was the implementation of modern videostereophotogrammetry in orthodontics and its use to capture facial movements in 4D. In contrast to previous attempts analysing facial expressions using markers on the patients’ face [4, 6, 7], static recordings of the movement at its maximal extent [31, 32], two-dimensional videography [33, 34], or the use of the facial acting coding system [8, 35], 4D stereophotogrammetry offers an objective and reliable method to measure facial movements. Static recordings at rest position and maximum extent bear the risk of unnatural freezing of the facial expression. Compared to 3D measurements, 2D analyses using videos underestimate the amplitude of facial motion by as much as 43% [36]. This discrepancy is particularly pronounced in the area of the lower face, which is usually in the focus of orthodontics. Marker-based tracking systems are criticised because the direct positioning of the markers on the patient’s face shows large variances and may affect the natural facial expressions [37]. Videostereophotogrammetry proved to be a feasible objective tool for assessing the impact of surgical interventions on facial movements [15], and demonstrated good to excellent reliability within the sample of this study.

A current disadvantage of videostereophotogrammetry is that the necessary cameras, lightning systems, computers, and software are expensive and bulky. However, portable low-cost 4D cameras already exist and make their way in clinical application [38, 39]. Big companies like Microsoft are working on these technologies trying to facilitate automatic landmark tracking, which will not only reduce costs of videostereophotogrammetry but also emphasise the impact of 4D measurements in the near future [40].

### Main findings in context of the existing evidence—results

The main finding was that orthodontic-surgical treatment increases the magnitude of the facial expression maximum smile by approximately 13% (= 2 mm), while it decreases that of the facial expression lip purse by approximately 11% (= 1 mm). The parameters shape change, time, and symmetry of the movement demonstrated no relevant changes from T0 to T1. The novelty of videostereophotogrammetry makes it difficult to compare these results with existing evidence. There are first attempts to integrate the dynamics of facial motion in orthodontic-surgical research [6, 8, 17, 33]. However, the interpretation of the data is limited by small study samples, methodological limitations, and divergent results. In 13 patients with maxillary hypoplasia and Le Fort I osteotomy, Al-Hiyali and co-workers reported a reduction in the magnitude of facial expressions of approximately 23% [17]. Since the authors summarised the data for three facial expressions (maximum smile, lip purse, and cheek puff), it remains unclear whether the change in the magnitude of movement differed between maximum smile and lip purse like observed in the current sample. Furthermore, in accordance with our observations, they demonstrated no clinically relevant difference in the asymmetry score for patients without facial asymmetry. For three patients with skeletal class III, Nooreyazdan and co-workers reported greater upward movement for cheilion left and right during smiling and reduced lip movement during lip purse [6], which is in agreement with the present results. Johns and co-workers, who also observed an increase in facial movement while smiling in patients with maxillary advancement, postulated that the anterior movement of the maxilla lengthens the facial muscles resulting in increased mobility [33]. This might only explain the increased magnitude in maximum smile in part as we found no interaction between the surgical movements and the parameters of facial expressions. Maybe, the explanation for the increased smile is as simple as the patients prefer smiling and displaying their teeth after surgical correction of the dentofacial deformity more than before. Nevertheless, it can be speculated that the surgical change in the underlying skeletal support affects the mobility of the overlying soft tissues. How good this adaption succeeds and whether the dynamics of facial soft tissues have an impact on skeletal relapse remain to be elucidated and is an aspect for future studies using the novel method of 4D motion capture. However, no significant impact of surgical movements, type of surgical intervention, or skeletal morphology on soft tissue dynamics was observed in this preliminary study. This may be due to the small sample sizes, implying a comparably low statistical power, but indicates that the postsurgical adaptation is highly individual and prediction of postsurgical facial movements is currently not possible. In contrast, Nooreyazdan and co-workers found presurgical differences between patients with skeletal class II, open bite, and class III for the movement lip purse, while there were no differences for the movements smile, mouth opening, cheek puff, eye opening, eye closure, or grimace even though their sample size was even smaller than in the present study [6].

Furthermore, the question arises whether 1 or 2 mm (11–13%) changes in the magnitude of the facial expressions, like found in the present study, are clinically relevant. With regard to social interaction the interpretation of the data gets even more complex as culture and gender shapes our expectations about the intensity of facial expressions in the sense of emotions [41, 42]. In contrast to orthopaedics, where gait measures start to establish as potential disease biomarkers [43, 44], no references for an ideal facial movement exist in orthodontics. Therefore, efforts should be made to collect movement data in class I subjects and the integration of facial dynamics in diagnostics should be driven forward to achieve patient-centred comprehensive treatment plans. Until then, differences of 1–2 mm should be judged as relevant because studies assessing the activity of other muscles of the skull, e.g. the dimension of masticatory muscles or jaw movement, often show pre-/postsurgical differences of 1–2 mm [45, 46].

### Limitations

The participants of the study were not operated on by the same surgeon, but all surgeries were performed at a single centre following the same stringent protocol. Since a multi-centre study revealed that even different surgeons at different centres obtain similar accuracy when following a virtual treatment plan [47], it can be assumed that the surgical results within this study were reproducible and representative for orthodontic-surgical interventions.

It is possible that the patients’ compliance, the intensity of care provided by the orthodontic-surgical treatment team, and subsequently the speed of post-surgical recovery were influenced by the fact that the patients were part of a clinical trial. Because of that Hawthorne effect, the participants in the present study might show increased facial expressiveness.

The observed changes in maximum smile and lip purse were rather small. Due to the novelty of the method, there are no reference values which indicate what amount of change should be considered clinical relevant and what extent of change is detected by orthodontists, surgeons, laypersons, or the patients themselves. Future studies are needed to generate norm values.

### Generalisability

The results of this trial were from a single specialist clinic in Germany, which might reduce the applicability to other clinical settings. However, the eligibility criteria include most patients undergoing orthodontic-surgical treatment and the followed surgery protocol is widely used and well established.

This study did not have the purpose to investigate gender differences in orthodontic surgical treatment and gender distribution within the study sample was not equal. This reflects the real clinical situation since women are more likely to accept surgical intervention than men [48–50]. The results may be generalised to a similar population with indication for orthodontic-surgical treatment and similar gender distribution.

### Further research

The focus of this study was the integration of videostereophotogrammetry in orthodontic-surgical treatment. To fully understand the effect of orthodontic-surgical treatment on facial expressions, long-term observations and extended study periods are needed. Baseline assessment was performed after orthodontic decompensation with fixed appliances in situ, which might have affected facial movements. Future studies should consider recruitment of patients with severe malocclusions prior orthodontic treatment. The follow-up of 4 months post-surgical in this study was chosen because the swelling has usually subsided by that time [51, 52] and the fixed appliance is still in place. This allowed a comparison of the pre- and post-surgical data without considering an influence of the orthodontic appliance. Since we expected an average post-surgical orthodontic treatment time of 5 to 6 months [53, 54], T1 was set at 4 months post-surgical to reduce dropouts. However, studies on changes of masticatory performance following orthodontic-surgical treatment indicate that an observation period of up to 5 years is necessary to show all effects induced by the intervention since the muscles of mastication need time to regain full strength [55, 56]. Whether this also applies to the facial muscles has not yet been investigated and further studies with an extended follow-up period are required.

## Conclusions

The present study implemented the novel approach of 4D motion capture using videostereophotogrammetry in orthodontic research. Within the limitations of this clinical trial, the following conclusion can be made:

1. Implementation of videostereophotogrammetry in orthodontic-surgical treatment is feasible and allows the evaluation of facial movements.
2. The magnitude of the expression maximum smile increases while that of lip purse decreases.
3. This change in facial movements following orthodontic-surgical treatment was observed independently of skeletal classes, surgical movements, or type of surgical intervention in this preliminary study.

### Supplementary Information

Below is the link to the electronic supplementary material.

Supplementary file1 (MP4 39959 KB)

## Data Availability

The data that support the findings of this study are not openly available due to reasons of sensitivity and are available from the corresponding author upon reasonable request.
